# Heat‐not‐burn tobacco (IQOS), oral fibroblasts and keratinocytes: cytotoxicity, morphological analysis, apoptosis and cellular cycle. An in vitro study

**DOI:** 10.1111/jre.12888

**Published:** 2021-05-21

**Authors:** Stefano Pagano, Paolo Negri, Maddalena Coniglio, Stefano Bruscoli, Alessandro Di Michele, Maria Cristina Marchetti, Chiara Valenti, Angela Gambelunghe, Luca Fanasca, Monia Billi, Stefano Cianetti, Lorella Marinucci

**Affiliations:** ^1^ Department of Medicine and Surgery Odontostomatological University Centre: Chair Prof. Stefano Cianetti University of Perugia Perugia Italy; ^2^ Department of Medicine and Surgery Section of Pharmacology University of Perugia Perugia Italy; ^3^ Department of Physics and Geology University of Perugia Perugia Italy; ^4^ Department of Medicine and Surgery Section of Occupational Medicine, Respiratory Diseases and Toxicology University of Perugia Perugia Italy; ^5^ Department of Molecular Medicine Biotechnology University of Naples Federico II Naples Italy; ^6^ Department of Medicine and Surgery Section of General Pathology University of Perugia Perugia Italy; ^7^ Department of Medicine and Surgery Section of Biosciences and Medical Embryology University of Perugia Perugia Italy

**Keywords:** apoptosis, cytotoxicity, fibroblast, keratinocyte, smoking devices

## Abstract

**Objectives:**

The aim of this work is to investigate the biological effects of IQOS smoking on human gingival fibroblasts and human keratinocytes analysing cell viability, morphology, migration, apoptosis and cell cycle.

**Background:**

Electronic cigarettes and tobacco heating systems have been marketed to reduce smoking damages caused by combustion.

**Methods:**

Human gingival fibroblasts and human keratinocytes viability was determined by a colorimetric assay measuring mitochondrial dehydrogenase activity (MTT assay); after an in vitro exposure of 24 h, cell morphology was analysed with scanning electron microscope and cell migration was tested by Scratch assay, a method to mimic the migration of the cells during wound healing in vivo. Apoptosis and cell cycle were analysed with flow cytometry, and the expression of related genes (p53, Bcl2, p16 and p21) was indagated using real‐time polymerase chain reaction.

**Results:**

IQOS extracts increased both cell viability (23%‐41% with fibroblasts and 30%‐79% with keratinocytes) and migration. No morphological alterations were observed. IQOS extracts did not induced an increase in cell death, but rose the number of S‐ and G2/M‐phase cells. IQOS extracts also significantly increased p53 expression by fibroblasts (undiluted and 6.25% dilution, 2‐ and 3.6‐fold higher, respectively) and reduced both Bcl2 (about two‐ and fivefold, respectively) and p21 expressions (about twofold with both extracts), while on keratinocytes both undiluted and 6.25% dilution extracts increased Bcl2 expression (about four‐ and threefold higher, respectively) and reduced p53 expression (about two‐ and fivefold, respectively).

**Conclusion:**

IQOS smoke seemed to induce proliferation as highlighted by a viability assay, and migration and cell cycle analysis. The increased cell proliferation induced by IQOS devices must be carefully investigated for its possible clinical effects on oral cell populations.

## 1 INTRODUCTION

Tobacco smoking is one of the most serious public health problems the world has ever faced. Despite over 8 million deaths per year that can be directly attributed to it, there are still about 1.3 billion smokers worldwide.^1^ Smoking is the main cause of mortality due to cardiovascular diseases like atherosclerosis and its consequences,^2^ respiratory disorders such as chronic obstructive bronchopathy (COPD),^3^, ^4^ chronic bronchitis and pulmonary emphysema,^5^ bone disorders such as osteoporosis^6^, ^7^ as well as cancer, particularly lung, bladder, liver, breast and oral malignancies.^8^, ^9^ Consequently, governments worldwide have promoted anti‐smoking campaigns and laws. Many devices were developed to induce smoking cessation like, for example, nicotine patches and chewing gums which, as they did not replicate the smoking ritual, often failed in their aim.^10^ Despite this, electronic cigarettes and the IQOS cigarette are still widespread today. As an alternative to tobacco smoking, these devices are assumed to be less harmful but their real effects are still not known.

The IQOS battery‐powered heated tobacco product (HTP), which is manufactured by Philip Morris International, was launched in 2014 and approved by the US Food and Drug Administration (FDA) for the US market in April 2019. Today it is found on 51 markets in Europe, South America and Asia.^11^ Resembling a traditional cigarette, the battery‐powered IQOS device contains a tobacco cartridge of about 3 cm in length with or without a filter. The cartridge is inserted into a self‐heating cylinder in the portable battery which, thanks to aluminium foil, heats it up to a temperature of about 350℃.^12^ The kit also contains a battery charger.

The tobacco cartridges consist of:

- Tobacco, treated with glycerine, with 0.5 mg of nicotine
- External paper covering thin aluminium foil around the tobacco
- A cooling filter in biopolymer
- A hollow acetate cellulose mouthpiece around the filter.

The main advantage of the IQOS device lies in its non‐combustion of tobacco which is heated to much lower temperature than in traditional cigarettes. In fact, as most of the 0.3 mg–0.8 mg of nicotine and 0.0005 mg of tar burns in a normal cigarette, about 20% is absorbed into the body.^13^, ^14^ According to the manufacturers, lack of combustion products and toxic smoke in the IQOS device eliminates or reduces most harmful tobacco‐related substances and health risks.^15^ Conversely, several studies observed that the IQOS aerosol contained substances derived from pyrolysis and that thermogenic degradation of the tobacco cartridge was similar to traditional cigarette smoke.^15^ Toxic components of cigarette smoke such as tar, nicotine, carbonyl compounds (formaldehyde, acrolein, acetaldehyde) and nitrosamines were detected in the IQOS aerosol, thus potentially constituting a risk to human health.^16^ Indeed, one analysis showed that the IQOS emissions contained organic acids, nicotine and aldehydes like acetaldehyde, formaldehyde and acrolein, although in lower concentrations than in traditional cigarettes.^17^ Some studies even showed that menthol‐flavoured IQOS contained even higher concentrations of these harmful components.^18^

To date, the very few scientific studies who have investigated IQOS toxicity have mainly focused on the IQOS device itself and on comparing its aerosol composition with traditional and electronic cigarettes.^19^ Several controversies have emerged over the quantity of harmful substances that IQOS releases, whether its emission should be classified as an aerosol or smoke and, if smoke, the effects of passive smoking.^20^, ^21^ In vitro studies demonstrated that the IQOS aerosol toxicity was intermediate between electronic and traditional cigarettes. Furthermore, it damaged respiratory tract cells, the only cells that have been investigated to date.^22^, ^23^

As the oral cavity is the first anatomic structure to come into contact with cigarette smoke and the IQOS aerosol, an analysis seemed required of their effects on specific oral cell populations. The present in vitro study assessed the biological effects of IQOS cigarette smoking on oral fibroblasts and keratinocytes. Specifically, viability was determined by the MTT assay, morphology by SEM analysis and migration by the scratch wound assay. Apoptosis and cell cycle were analysed by flow cytometry. Expression levels of p53, Bcl2, p16 and p21 genes were assessed by RT‐PCR.

The first null hypothesis is that the biological effects caused by IQOS devices are similar to those generated by traditional cigarette.

The second null hypothesis is that the results obtained from this in vitro study do not allow to advance suppositions on damages generated by IQOS device.

## 2 MATERIALS AND METHODS

### 2.1 Study design

The aim of this study is to compare the effects of the IQOS aerosol on exposed *vs* non‐exposed human gingival fibroblasts and human keratinocytes.

### 2.2 Test material

IQOS Heat‐not‐burn kits (^©^Phillip Morris Products S.A., Switzerland) and cartons of IQOS Marlboro Yellow Heat Sticks (HEETS) (^©^Phillip Morris Brands Srl, Italy) were purchased in Perugia, Italy. The main features are described in Table 1.

**TABLE 1:** Principal components and manufacturers of IQOS device

Name	Components	Description/Features	Image	Production
Heat‐not‐burn IQOS	Holder	Height 93.6 mm Diameter 15.04 mm Weight 20 g	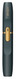	© Philip Morris Products SA.
	Portablebatterycharger	Height 112.5 mm Diameter 21.86 mm Width 51.2 mm Weight 100 g Capacity2900 mAh	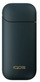	
HEETS Yellow Label	TobaccoStick	Height 45 mm Diameter 7 mm Tobacco 0.3 g with 0.5 mg of nicotine	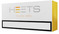	

### 2.3 Cell culture

BSCL138 human gingival fibroblasts (IZSLER, Brescia, Italy) were grown as monolayer cultures in sterile polystyrene T‐75 flasks (Thermo Fisher Scientific, Waltham, MA USA) containing Eagle's minimum essential medium (MEM, Thermo Fisher Scientific, Waltham, MA USA) supplemented with 10% foetal bovine serum (FBS, Thermo Fisher Scientific, Waltham, MA USA), penicillin (10 000 U/mL), streptomycin (10 000 μg/mL) and 25 μg/mL amphotericin B as anti‐fungal agent (Thermo Fisher Scientific, Waltham, MA USA).

BSCL 143 human keratinocytes (IZSLER, Brescia, Italy) were grown as monolayer cultures in sterile polystyrene T‐75 flasks (Thermo Fisher Scientific, Waltham, MA USA) containing Dulbecco's modified Eagle's medium (DMEM, Thermo Fisher Scientific, Waltham, MA USA) supplemented with 7% foetal bovine serum (FBS, Thermo Fisher Scientific).

Fibroblasts and keratinocytes were maintained in a humidified incubator at 37°C with 5% CO_2_ with twice weekly medium changes and monitored under a phase contrast Leitz inverted microscope. Upon 80% confluence (logarithmic growth phase), cells were detached with a mixture of 0.25% trypsin and 0.02% ethylenediaminetetraacetic acid (EDTA). After 1:1 dilution in Trypan Blue Dye (10 μL of cells and 10 μL of Trypan Blue), cells were counted in a Countess Automated Cell Counter (Thermo Fisher Scientific, Waltham, MA USA) and plated as described below. All tests were performed between the seventh and ninth subculture.^24^

### 2.4 Preparation of Cigarette IQOS Extract

IQOS extract was prepared by the modified Carp and Janoff method.^25^ Using a peristaltic pump, the following puff protocol was established: 4 s puff duration, every 30 s, with a 55 mL volume per puff. Ten puffs represented one smoking session lasting 318 s. The IQOS aerosol from one Yellow Heat Stick was driven into 20 mL of preheated culture media (MEM and DMEM), providing fresh 100% extract. The pH was adjusted to 7.4 and filtered through a 0.22 µm filter (Merck Millipore, Germany). The extract was serially diluted as following: twofold (50%), fourfold (25%), eightfold (12.5%), 16‐fold (6.25%) and 32‐fold (3.125%). Each dilution was done by volume in media. Negative controls were cells treated with fresh culture media (MEM and DMEM). The designed smoke system does not generate thermal alterations on the culture media.

### 2.5 Cytotoxicity assay (MTT)

Human gingival fibroblasts and human keratinocytes were seeded (10 000 cells/well) on optical clear 96‐well flat bottom microtitre plates (Thermo Fisher Scientific, Waltham, MA USA) and incubated for 24 h a 37°C in 5% CO_2_. Subsequently, the culture medium was discarded and replaced with 100 μL of diluted (from 50% to 3.125%) or undiluted extracts. Control groups were treated with fresh culture medium. Cell cultures were incubated for 24 h at 37°C in 5% CO_2_.

Cytotoxicity was assessed by a colorimetric assay measuring mitochondrial dehydrogenase activity.

Reduction of the soluble tetrazolium salt, 3‐[4,5‐dimethyl‐2‐thiazolyl]‐2–5‐diphenyl‐2H tetrazolium bromide (MTT, Sigma Chemical Co., St. Louis, MO, USA) to a formazan precipitate, causes a yellow‐to‐purple colour change.^26^ After treatment, 10 μL of MTT solution (5 mg/mL) were added to each well. Plates were covered and incubated for 4 h at 37°C. MTT‐derived formazan crystals were dissolved by adding dimethyl sulphoxide (100 µl/well; DMSO, Sigma Chemical Co., St. Louis, MO) under gentle shaking for 30 min. Absorbance was measured at 570 nm using an automatic microplate spectrophotometer reader (Bio‐Rad, Model 680 XR, CA).

According to ISO 10993‐5,^27^ fewer viable cells resulted in decreased mitochondrial enzyme activity (succinic dehydrogenase, SDH) which directly correlated with the amount of blue–violet formazan produced by the tetrazolium salt reduction. Absorbance values in control and treated groups were compared. Cell viability was calculated according to the following formula using optical density (OD): % cell viability = (OD ratio of treated group/OD of control group) × 100.

### 2.6 Morphological analysis (SEM)

To determine the effects of extracts on cell morphology, human gingival fibroblasts and human keratinocytes were grown on a cover glass (12 mm diameter × 0.15 mm depth, Exacta Optech Lab Center SpA, Modena, Italy) in MEM supplemented with 10% FBS, antibiotics and anti‐fungal agent and DMEM supplemented with 7% FBS, respectively, for 24 h at 37°C in 5% CO_2_. The culture medium was discarded and replaced with 400 μL of 6.25% diluted or undiluted extracts. Control groups were treated with fresh culture medium. Cell cultures were incubated for another 24 h at 37°C in 5% CO_2_. Cells were thrice washed with PBS, fixed in 2.5% glutaraldehyde in phosphate‐buffered saline PBS for 1 h at room temperature, thrice washed with distilled water, dehydrated stepwise in ethanol and dried at room temperature.^28^ After critical point drying, using the Freon method, samples were splutter‐metallized with chromium (8 nm) and viewed under a Field Emission Gun SEM LEO 1525 (ZEISS, Jena Germany). The acceleration voltage was 5 KV and images were obtained using an in‐lens detector. Magnifications were: 500×, 2500×, 5000× and 10 000×.

### 2.7 Scratch assay

To investigate fibroblast and keratinocyte migration, cells were plated on 6‐well flat bottom microtitre plates (Thermo Fisher Scientific) and grown in 2 mL medium. At 90% confluence, medium was removed and, using a sterile P‐200 pipette tip, a straight scratch was done along the monolayer in the well centre, as described elsewhere.^29^ Cellular debris was gently removed with Dulbecco's phosphate‐buffered saline (PBS) and cultures were exposed to undiluted (100%) and 6.25% diluted extracts. Wound closure images were obtained at 0 h, 17 h, 24 h for human gingival fibroblasts and at 0 h, 20 h, 40 h, 64 h and 70 h for human keratinocytes, using a conventional phase‐contrast microscope (Olympus, Tokyo, Japan). Photographs at 200× magnification provided migration and morphology profiles.

### 2.8 RNA isolation and RT‐PCR analysis

To analyse apoptosis and cell cycle genes, human gingival fibroblasts and keratinocytes were seeded (1 × 10^5^ cells/ml) in 6‐well flat bottom microtitre plates (Thermo Fisher Scientific). At confluence, cells were treated with 6.25% diluted and undiluted extracts or fresh medium (control groups) for 24 h.

Total RNA was isolated as described elsewhere.^29^ Briefly, RNA from control and treated fibroblasts/keratinocytes was isolated using a total RNA purification kit (Thermo Fisher Scientific) and quantified by reading the OD at 260 nm on a BioPhotometer (Eppendorf, Milano, Italia). Using ABM (Richmond, Canada), 1 μg total RNA was subjected to reverse transcription (RT) in a final volume of 20 μL. Real‐time PCR was performed using 2 μL cDNA from the RT reaction. The primer sequences of each gene are listed in Table 2. Primers were designed with PERL primer software using NCBI EntrezGene reference sequences as template and synthesized by Thermo Fisher Scientific. Real‐time PCR was carried out in an Mx3000P cycler (Stratagene, Amsterdam, the Netherlands) using FAM for detection and ROX as reference dye. One‐step PCR was performed in 25 mL of Brilliant SYBR(r) Green QPCR Master Mix (Stratagene, Amsterdam, the Netherlands), according to the manufacturer's instructions. At each annealing step, product formation was monitored with the fluorescent double‐stranded DNA binding dye SYBR(r) Green. The relative expression level of the housekeeping gene glyceraldehide‐3‐phosphate dehydrogenase (GAPDH) was used to normalize marker gene expression in each sample. Immediately after PCR, a melting curve was undertaken by raising the incubation temperature from 55° to 95°C to confirm amplification specificity. Results were expressed as fold change relative to untreated control values; all values were computed with the MxPro QPCR Software (Stratagene).

**TABLE 2:** Primer sequences used for RT‐PCR analysis

mRNA	Sequences (5’‐3’)	Product (bp)
GAPDH	Fw: TGGTATCGTGGAAGGACTCATGAC Rv: ATGCCAGTGAGCTTCCCGTTCAGC	188
p53	Fw: GGACCTGATTTCCTTACTG Rv: TGAATCTGAGGCATAACTG	248
Bcl2	Fw: AGATGTCCAGCCAGCTGCACCTGAC Rv: AGATAGGCACCCAGGGTGATGCAAGCT	366
p21	Fw: TGGAGACTCTCAGGGTCGAA Rv: GACTGCAGGCTTCCTGTGG	118
p16	Fw: CCCAACGCACCGAATAGTTA Rv: CACCAGCGTGTCCAGGAA	173

### 2.9 Apoptosis and cell cycle analysis

Apoptosis and cell cycle analysis were assessed by flow cytometry as previously described.^29^ Briefly, 24 h after treatment with undiluted and 6.25% diluted extracts for 24 h, human gingival fibroblasts and keratinocytes were harvested, re‐suspended in 0.5 mL hypotonic propidium iodide (PI) solution (50 μg/mL propidium iodide in 0.1% sodium citrate plus 0.1% TritonX‐100) and analysed by flow cytometry using Coulter Epics XL‐MCL Flow Cytometer (BeckmanCoulter). Data were analysed using FlowJo software (TreeStar).

### 2.10 Statistical analysis

Figures report the mean ± SD (standard deviation) of three independent experiments performed in quintuplicate. One‐way analysis of variance (ANOVA) was performed using GraphPad Prism 5.01 software (Prism, CA, USA). *p* values of <.05 were considered statistically significant.

## 3 RESULTS

### 3.1 Cytotoxicity assay (MTT)

**FIGURE 1:**
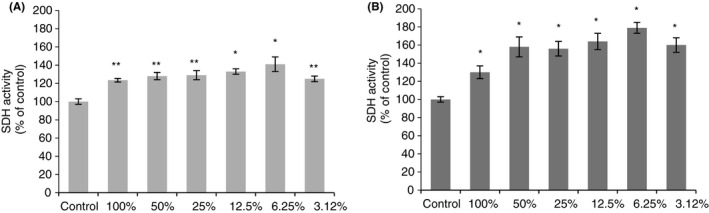
Effects of IQOS extracts (diluted and undiluted) on human gingival fibroblasts (A) and human keratinocytes (B) using the MTT assay. The results for each extract are expressed as the percentage of SDH activity compared with the control (100%). The values represent the mean ± SD of three independent experiments performed in quintuplicate for each sample. Differences vs. control: *p < .001; **p < .05

At all dilutions, the IQOS extract increased human gingival fibroblast viability by a mean of 30% (range 23% with undiluted extract – 41% with the 6.25 dilution). At 100%, 50%, 25% and 3.12%, dilutions, significance reached *p* < 0.05 which dropped to *p* < .001 with 12.5% and 6.25% dilutions (Figure 1A).

At all dilutions, the IQOS extract increased human keratinocyte viability a mean of 60% (range 30% with undiluted extract ‐ 79% with the 6.25% dilution) (*p* < .001) (Figure 1B).

Viability increases in both cell lines were due to a proliferation stimulus. Consequently, the following experiments used only undiluted extract and 6.25% diluted.

### 3.2 Morphological analysis (SEM)

**FIGURE 2:**
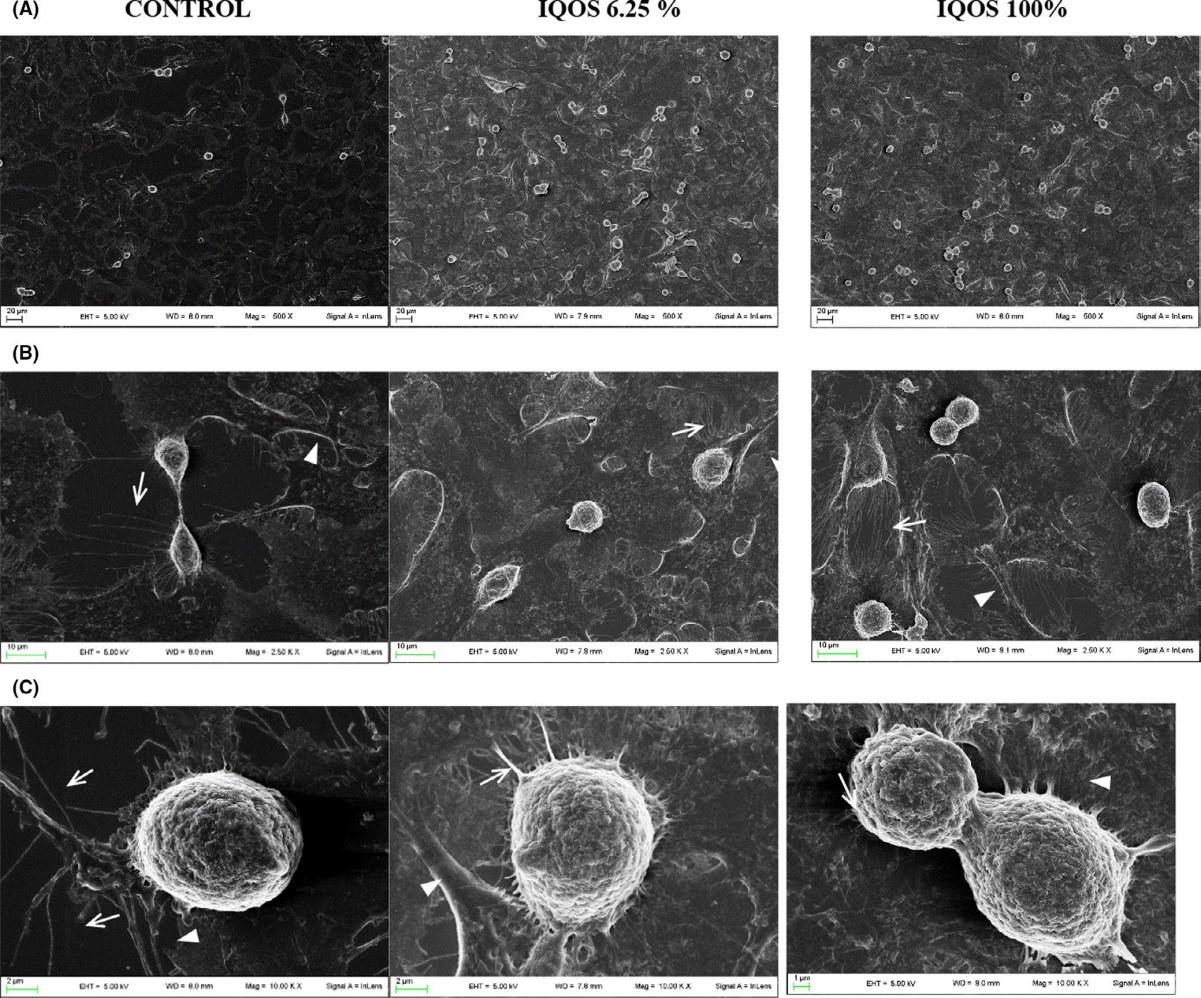
Effects of IQOS extracts on human gingival fibroblast morphology. Scanning electron microscopic (SEM) micrographs of untreated human gingival fibroblasts (control) or fibroblasts exposed to undiluted and diluted to 6.25% extracts with different magnification (A 500×, B 2500×, C 10 000×) after 24 h of culture. Filopodia indicated by arrows and lamellipodia by arrow heads

Besides confirming cell proliferation (Figure 2A), SEM analysis showed undiluted and 6.25% diluted IQOS extracts induced only cell surface corrugation in gingival fibroblasts (Figure 2B, C) compared with control cells. Neither IQOS extract impacted on strong fibroblasts substrate adherence. As in control cells, several filopodia and lamellipodia were observed, indicating adhesion and migration (Figure 2B, C).

**FIGURE 3:**
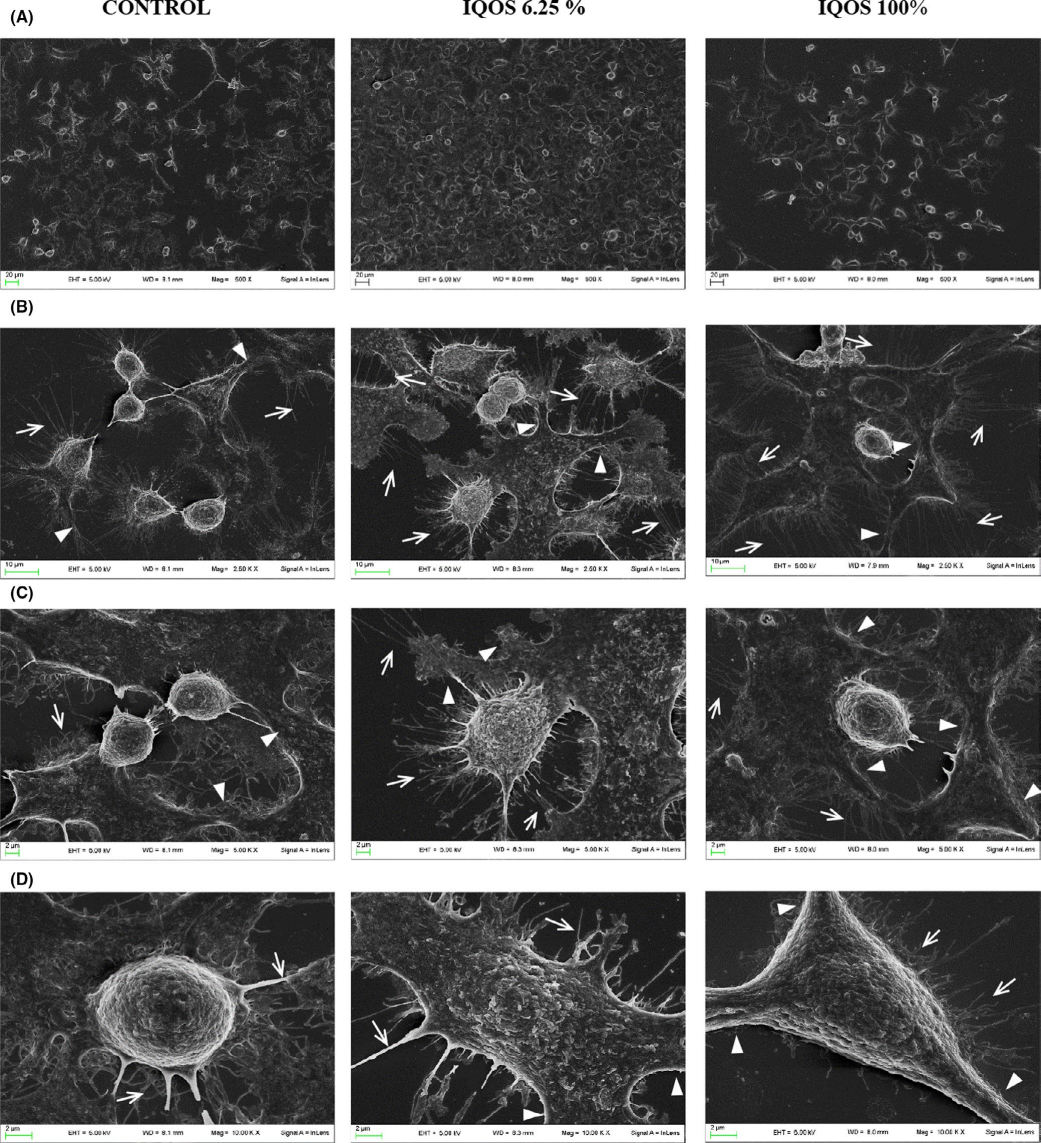
Effects of IQOS extracts on human keratinocyte morphology. Scanning electron microscopic (SEM) micrographs of untreated human keratinocytes (control) or keratinocytes exposed to undiluted and diluted to 6.25% extracts with different magnification (A 500×, B 2500×, C 5000×, D 10 000×) after 24 h of culture. Filopodia indicated by arrows and lamellipodia by arrow heads

Compared with controls, keratinocyte proliferation was greater with the 6.25% diluted extract than with the undiluted (Figure 3A) and more filopodia and lamellipodia were observed (Figure 3B, C) which were long and fine after exposure to undiluted extract and short and thick after exposure to the 6.25% diluted (Figure 3D).

### 3.3 Scratch test

**FIGURE 4:**
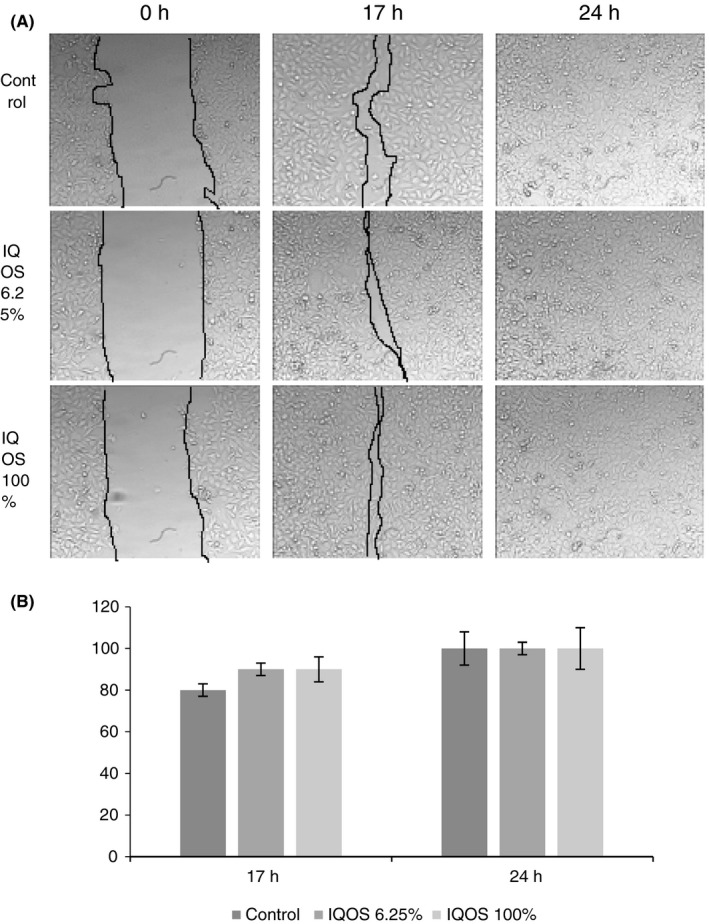
Effect of undiluted (100%) and diluted to 6.25% IQOS extracts on human gingival fibroblast migration in the wound‐healing migration assay. (A) Representative phase‐contrast images of the wounds were taken at 0 h, 17 h and 24 h (200× magnification). (B) Quantification of the percentage of closed wound area calculated by tracing the border of the wound using ImageJ software. Data represent the mean ± SD of three independent experiments

Both IQOS extracts induced a similar, non‐significant increase in migration in gingival fibroblasts compared with controls. After 24 h, all wounds were closed (Figure 4A, B).

**FIGURE 5:**
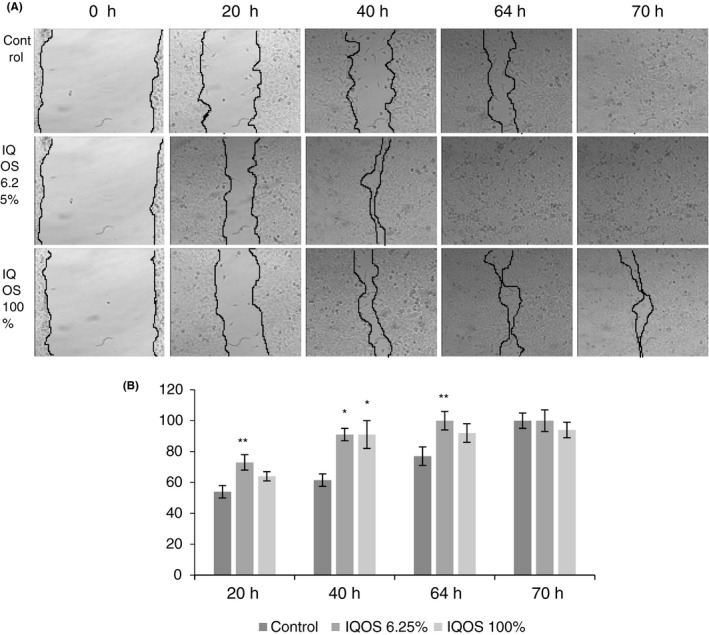
Effect of undiluted (100%) and diluted to 6.25% IQOS extracts on human keratinocyte migration in the wound‐healing migration assay. (A) Representative phase‐contrast images of the wounds were taken at 0 h, 20 h, 40 h, 64 h and 70 h (200× magnification). (B) Quantification of the percentage of closed wound area calculated by tracing the border of the wound using ImageJ software. Data represent the mean ± SD of three independent experiments. Differences vs. control: *p < .001; **p < .05

Migration patterns varied with dilution in keratinocytes. The undiluted extract stimulated migration for 40 h after which migration slowed and, like controls, the wound remained open at 70 h. The 6.25% diluted extract stimulated migration more strongly and wound closure was observed at 64 h (Figure 5A, B).

### 3.4 Apoptosis and cell cycle analyses

**FIGURE 6:**
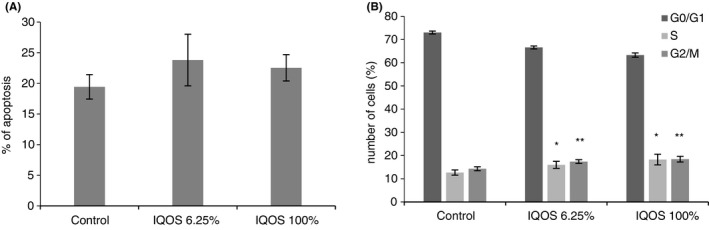
Effect of cigarette IQOS extracts on apoptosis (A) and on cell cycle (B). Human gingival fibroblasts were treated with undiluted and diluted to 6.25% extracts for 24 h. Cells were collected and stained with PI and analysed by flow cytometry for percentage of apoptotic cells (a) and for percentage of cell in different phases of cell cycle (B). The values represent the mean ± SD of three independent experiments performed in triplicate for each extract. Differences vs. control: *p < .001; **p < .05

**FIGURE 7:**
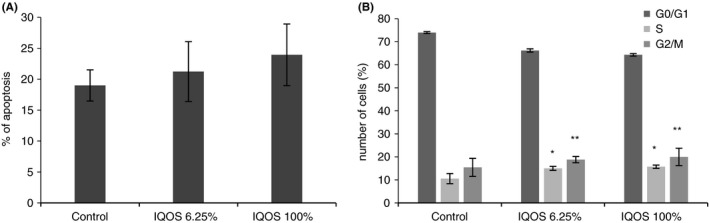
Effect of cigarette IQOS extracts on apoptosis (A) and on cell cycle (B). Human keratinocytes were treated with undiluted (100%) and diluted to 6.25% extracts for 24 h. Cells were collected and stained with PI and analysed by flow cytometry for percentage of apoptotic cells (A) and for percentage of cell in different phases of cell cycle (B). The values represent the mean ± SD of three independent experiments performed in triplicate for each extract. Differences vs. control: *p < .001; **p < .05.

Flow cytometry detected no significant difference in fibroblast and keratinocyte apoptosis 24 h after exposure to undiluted and 6.25% diluted IQOS extracts (Figures 6A and 7A). Both extracts significantly increased in the percentage of each cell in the S (*p* < .001 for both) and G2/M (*p* < .05 for both) cell cycle phases (Figures 6B and 7B).

### 3.5 Apoptosis and cell cycle related gene expression

**FIGURE 8:**
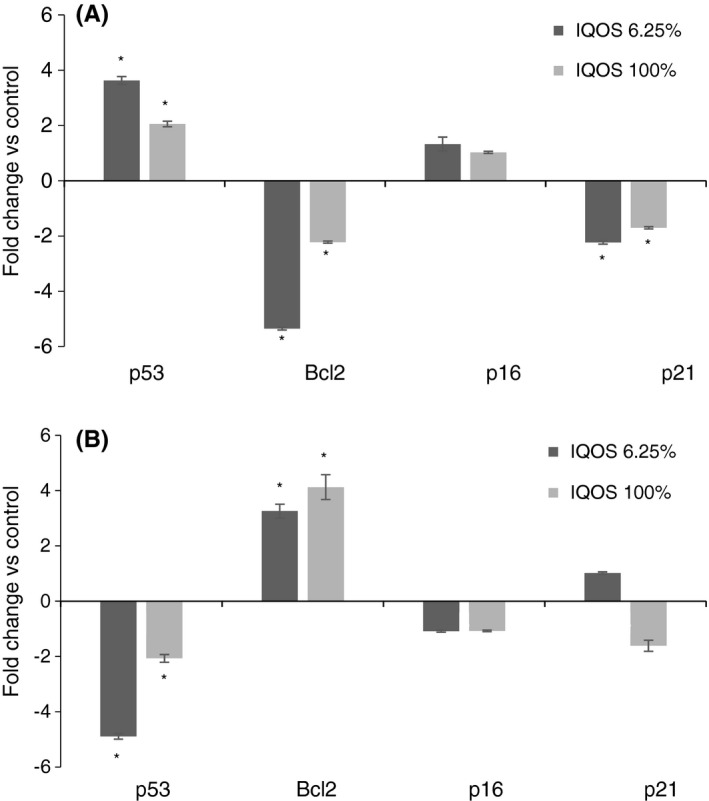
Effect of undiluted (100%) and diluted to 6.25% IQOS extracts on apoptosis and cell cycle related gene expression (*p53*, *Bcl2*, *p16* and *p21)* evaluated by RT‐PCR at 24 h on human gingival fibroblasts (A) and human keratinocytes (B). The results for each extract are expressed as fold change in GAPDH normalized mRNA values. The values represent the mean ± SD of three independent experiments performed in triplicate for each sample. Differences vs. control: *p < .001

In human gingival fibroblasts, undiluted (100%) IQOS cigarette extract and 6.25% diluted extract significantly increased p53 expression, by approximately two‐ and 3.6‐fold, respectively, compared with controls (*p* < .001), decreased Bcl2 expression levels by approximately two‐ and fivefold, respectively (*p* < .001), and p21 levels by approximately twofold both (*p* < .001), while p16 expression appeared unchanged (Figure 8A).

In human keratinocytes, undiluted (100%) IQOS cigarette extract and 6.25% diluted extract significantly downregulated p53 expression by approximately two‐ and fivefold, respectively (*p* < .001), and increased Bcl2 expression four‐ and threefold, respectively, compared with controls (*p* < .001). p16 and p21 expression remained unchanged (Figure 8B).

## 4 DISCUSSION

In the human body smoke first encounters the oral cavity and comes directly into contact with the oral mucosa. Through dissolution in saliva its components are absorbed by the oral epithelium and about 100 of the 5000 chemical substances are potentially toxic. Moreover, smokers are at greater risk of more severe, non‐responsive periodontitis than non‐smokers.^30^ Most in vitro investigations reported cigarette smoking decreased viability and proliferation of oral cell populations,^31^, ^32^ cellular migration,^33^, ^34^ induced morphological modifications^35^, ^36^ and production of inflammatory mediators^37^, ^38^ as well as cell cycle blocking, arrest and apoptosis.^39^ All of these effects appear to be dose‐ and time‐dependent.^35^, ^40^, ^41^ Another important aspect about cigarette smoking toxicity is heating that could determine prolonged oral mucosa irritation, tissues thickening and increase in keratinization, with potential malignant transformation of tissues.^42^ The heat also causes changes in the hard palate such as inflammation of tissue and irritation of the minor salivary glands that present inflamed ducts and in the lower lip with the development of actinic cheilitis or lip cancer.^43^

Early studies of the IQOS device focused on aerosol composition, highlighting it contained several potentially harmful components though at lower concentrations than traditional cigarette smoke. Gas chromatography–mass spectrometry (GC‐MS) detected 62 volatile compounds, including carbonyls like propanal, acrolein, 3‐methylbutanal, diacetyl and 2,3‐pentanedione, heavy metals, flavouring chemicals, acrolein, diacetyl and 2,3‐pentanedione, all of which are highly toxic and constitute a health risk, particularly an oral health risk.^41^ As the few in vitro studies investigated the effects of the IQOS device on pulmonary cells^22^, ^23^ or human osteoclasts,^12^ the present study focused on human keratinocytes and human gingival fibroblasts.

The first difficulties we had to overcome were capturing all the aerosol components given their volatility and administering them to cell cultures. We designed a smoke production mechanism consisting of a vacuum pump that sucked the IQOS aerosol into a flask containing culture medium which absorbed all its components. It was needed because, even though cigarette toxicology studies used smoke machines with fixed smoke conditions, exposure protocols had been shown to be sub‐optimal because cigarette aerosols were altered.^44^, ^45^ Another difficulty lay in testing the same quantity of aerosol quantity as found in IQOS consumers. The present study opted for an exposure protocol corresponding to a smoking time of 5 min and 18 s, which is the time needed to smoke one stick containing 5 mg nicotine, bearing in mind that nicotine content is always constant in IQOS sticks, unlike traditional and e‐cigarettes.

This preliminary study observed the IQOS aerosol appeared to be less toxic than traditional cigarette smoke. Although the aerosol did not damage the oral cavity, it stimulated cell proliferation, as shown by more prolonged and intense S and G2/M cell cycle phases, greater viability and migration and more adhesive features in keratinocyte morphology. Surprisingly, the expression of genes related to apoptosis and the cell cycle did not fully support these findings.

All aerosol concentrations increased vitality in human fibroblasts and more markedly in keratinocytes as assessed, in accordance with ISO 10993‐5 recommendations, through mitochondrial activity, concurring with reports using assays such as LDH or Neutral red in addition to MTT.^44^, ^46^ IQOS was reported to be less cytotoxic than traditional cigarettes in human bronchial epithelial cells^23^ and had no effect on the viability of primary human osteoblasts and immortalized human mesenchymal stem cells after short‐ (48 h) and long‐term (14 and 21 days) treatments.^12^ Through proliferation, migration, synthesis of growth factors and extracellular matrix molecules, increased fibroblast proliferation could promote healing processes but, on the other hand, greater cellular metabolic activity could impact negatively on cell death and survival signalling pathways.^47^ Furthermore, dysregulation of succinate dehydrogenase, mitochondrial reductase and tumour suppressor genes, can promote uncontrolled cell proliferation, the mechanism underlying the onset of malignant tumours.^48^

Exposure to IQOS extracts did not alter the morpho‐functional status in fibroblasts but did increase filopodia and lamellipodia, cytoplasmic extensions in keratinocytes. The keratinocyte response to IQOS exposure might be an attempt to achieve greater stability by maintaining firm contact with the substrate and neighbouring cells. The IQOS extract did not affect human osteoblast morphology, as assessed by the length of primary cilia which are present during their differentiation.^12^

Innovative findings in the present study were that all IQOS concentrations induced a greater cell migration than control, as shown by the scratch test results which, as expected from the MTT results, was more marked in keratinocytes than in fibroblasts, particularly with the 6.25% IQOS extract.

IQOS exposure was associated with lack of apoptosis, a common form of cell death, which is in line with our findings on viability and migration patterns. Indeed, in human keratinocytes, expression of two key apoptosis‐related genes i.e. the anti‐apoptotic Bcl2 and pro‐apoptotic p53, was significantly upregulated and downregulated, respectively. Fibroblasts unexpectedly displayed the opposite pattern with p53 upregulation and Bcl2 downregulation. Prolonged active phases in the cell cycle were linked, respectively, to p21 expression which controls G0/G1 progression to the S phase and p16 expression which blocks the S phase.^49^ Expression of neither factor was altered in keratinocytes, while was significantly down‐regulated in fibroblasts.

These changes in gene expression indicated a response mechanism, whether defensive or aggressive still remains to be determined, had been activated by exposure to IQOS extracts. This response mechanism appears the complete opposite to what traditional smoking elicits in human cells as it is associated with high rates of cytotoxicity and apoptosis.^50^ Although many key apoptotic proteins that are activated or inactivated in apoptotic pathways have been identified, the molecular mechanisms underlying activation are not fully understood and are the focus of ongoing research.^51^ In an attempt to reconcile these apparently contradictory results, we might speculate that aldehyde, acreoline and heavy metal concentrations in the IQOS aerosol,^41^ could damage DNA by increasing strand breaks, triggering an initial mutagenicity/genotoxicity^52^ that was not yet detectable in the apoptosis rate. Assessment of tumour and proliferating genes will be needed to confirm this hypothesis. Alternatively, post‐transcriptional p53 gene modifications might prevent an increase in cell death due to apoptosis.

In conclusion, the clinical significance of the results of this preliminary investigation into the effects of the IQOS device are hard to discern. Our innovative system indicated that IQOS was not toxic for oral fibroblasts and keratinocytes as it neither modified survival nor morphology. Since it did impact on proliferation and the cell cycle further studies are needed to identify which components of the IQOS aerosol are involved. Another limitation of this study was restricting IQOS exposure to 24 h which precluded long‐term observations. So, it might be interesting to study a treatment repeated over time (chronic).

Even if the smoke system used in the study does not generate thermal alterations induced by the IQOS device, a further limitation of the study is represented by the need to evaluate the effects of the heating on the oral mucosa. Starting from these preliminary results our null hypotheses can be rejected.

## CONFLICT OF INTEREST

The authors declare that they have no conflict of interest.

## AUTHOR CONTRIBUTIONS

All authors contributed to the study conception and design. Material preparation, data collection and analysis were performed by Paolo Negri, Maddalena Coniglio, Stefano Bruscoli, Alessandro Di Michele, Maria Cristina Marchetti, Chiara Valenti, Angela Gambelunghe, Luca Fanasca, Monia Billi and Stefano Cianetti. The first draft of the manuscript was written by Stefano Pagano and Lorella Marinucci and all authors commented on previous versions of the manuscript. All authors read and approved the final manuscript.

## References

[1] Gorini G , Chellini E , Galeone D . What happened in Italy? A brief summary of studies conducted in Italy to evaluate the impact of the smoking ban. Ann Oncol. 2007;18(10):1620‐1622. 17804471 10.1093/annonc/mdm279

[2] Suzuki S , Otsuka T , Sagara K , et al. Effects of Smoking on Ischemic Stroke, Intracranial Hemorrhage, and Coronary Artery Events in Japanese Patients with Non‐Valvular Atrial Fibrillation. Int Heart J. 2007;58(4):506‐515. 10.1536/ihj.16-228 28701668

[3] Muñoz‐Lucas MA , Jareño‐Esteban J , Gutiérrez‐Ortega C , et al. Influence of Chronic Obstructive Pulmonary Disease on Volatile Organic Compounds in Patients with Non‐small Cell Lung Cancer. Arch Bronconeumol. 2020;S0300–2896(20):30006‐30015. 10.1016/j.arbr.2020.10.004 35373775

[4] Stabile AM , Marinucci L , Balloni S , Giuliani A , Pistilli A , Bodo M . Long term effects of cigarette smoke extract or nicotine on nerve growth factor and its receptors in a bronchial epithelial cell line. Toxicol In Vitro. 2018;53:29‐36. 30076938 10.1016/j.tiv.2018.07.020

[5] Mallampalli RK , Li X , Jang JH , et al. Cigarette smoke exposure enhances transforming acidic coiled‐coil‐containing protein 2 turnover and thereby promotes emphysema. JCI Insight. 2020;5(2):e125895. 10.1172/jci.insight.125895 PMC7098723 31996486

[6] Marinucci L , Bodo M , Balloni S , Locci P , Baroni T . Sub‐toxic nicotine concentrations affect extracellular matrix and growth factor signalling gene expressions in human osteoblasts. J Cell Physiol. 2014;229(12):2038‐2048. 24777817 10.1002/jcp.24661

[7] Marinucci L , Balloni S , Fettucciari K , Bodo M , Talesa VN , Antognelli C . Nicotine induces apoptosis in human osteoblasts via a novel mechanism driven by H_2_O_2_ and entailing Glyoxalase 1‐dependent MG‐H1 accumulation leading to TG2‐mediated NF‐kB desensitization: Implication for smokers‐related osteoporosis. Free Radic Biol Med. 2018;117:6‐17. 29355739 10.1016/j.freeradbiomed.2018.01.017

[8] Martínez Ú , Brandon TH , Sutton SK , Simmons VN . Associations between the smoking‐relatedness of a cancer type, cessation attitudes and beliefs, and future abstinence among recent quitters. Psychooncology. 2018;27(9):2104‐2110. 29785718 10.1002/pon.4774 PMC6156937

[9] Khowal S , Wajid S . Role of Smoking‐Mediated molecular events in the genesis of oral cancers. Toxicol Mech Methods. 2019;29(9):665‐685. 31345084 10.1080/15376516.2019.1646372

[10] IQOS . Phillip Morris International; 2017. https://www.pmi.com/smoke‐free‐products. Accessed 8 October 2017.

[11] Bar‐Zeev Y , Levine H , Rubinstein G , Khateb I , Berg CJ . IQOS point‐of‐sale marketing strategies in Israel: a pilot study. Isr J Health Policy Res. 2019;8(1):11. 30642383 10.1186/s13584-018-0277-1 PMC6330751

[12] Aspera‐Werz RH , Ehnert S , Müller M , et al. Assessment of tobacco heating system 2.4 on osteogenic differentiation of mesenchymal stem cells and primary human osteoblasts compared to conventional cigarettes. *World* . J Stem Cells. 2020;12(8):841‐856. 10.4252/wjsc.v12.i8.841 PMC7477651 32952862

[13] Churchill V , Weaver SR , Spears CA , et al. IQOS debut in the USA: Philip Morris International’s heated tobacco device introduced in Atlanta, Georgia. Tob Control. 2020;29(e1):e152‐e154. 32024772 10.1136/tobaccocontrol-2019-055488 PMC7416520

[14] Dautzenberg B , Dautzenberg MD . Le tabacchauffé: revue systématique de la littérature [Systematic analysis of the scientific literature on heated tobacco]. Rev Mal Respir. 2019;36(1):82‐103. 30429092 10.1016/j.rmr.2018.10.010

[15] Ratajczak A , Jankowski P , Strus P , Feleszko W . Heat not burn tobacco product‐a new global trend: impact of heat‐not‐burn tobacco products on public health, a systematic review. Int J Environ Res Public Health. 2020;17(2):409. 10.3390/ijerph17020409 PMC7014072 31936252

[16] O’Connell G , Wilkinson P , Burseg KMM , Stotesbury SJ , Pritchard JD . Heated tobacco products create side‐stream emissions: implications for regulation. J Environ Anal Chem. 2015;2:163.

[17] Ruprecht AA , De Marco C , Saffari A , et al. Environmental pollution and emission factors of electronic cigarettes, heat‐not‐burn tobacco products and conventional cigarettes. Aerosol Sci Technol. 2017;51(6):674‐684.

[18] Kogel U , Titz B , Schlage WK , et al. Evaluation of the Tobacco Heating System 2.2. Part 7: Systems toxicological assessment of a mentholated version revealed reduced cellular and molecular exposure effects compared with mentholated and non‐mentholated cigarette smoke. Regul Toxico Pharmacol. 2016;81(Suppl 2):S123‐S138. 10.1016/j.yrtph.2016.11.001 27818347

[19] Davis B , Williams M , Talbot P . IQOS: evidence of pyrolysis and release of a toxicant from plastic. Tob Control. 2019;28(1):34‐41. 29535257 10.1136/tobaccocontrol-2017-054104

[20] Auer R , Concha‐Lozano N , Jacot‐Sadowski I , Cornuz J , Berthet A . Heat‐Not‐Burn Tobacco Cigarettes: Smoke by Any Other Name. JAMA Intern Med. 2017;177(7):1050‐1052. 28531246 10.1001/jamainternmed.2017.1419 PMC5543320

[21] Bekki K , Inaba Y , Uchiyama S , Kunugita N . Comparison of Chemicals in Mainstream Smoke in Heat‐not‐burn Tobacco and Combustion Cigarettes. J UOEH. 2017;39(3):201‐207. 28904270 10.7888/juoeh.39.201

[22] Sohal SS , Eapen MS , Naidu VGM , Sharma P . IQOS exposure impairs human airway cell homeostasis: direct comparison with traditional cigarette and e‐cigarette. ERJ Open Res. 2019;5(1):159. 10.1183/23120541.00159-2018 PMC6368999 30775377

[23] Leigh NJ , Tran PL , O’Connor RJ , Goniewicz ML . Cytotoxic effects of heated tobacco products (HTP) on human bronchial epithelial cells. Tob Control. 2018;27(Suppl 1):s26‐s29. 30185530 10.1136/tobaccocontrol-2018-054317 PMC6252481

[24] Balloni S , Locci P , Lumare A , Marinucci L . Cytotoxicity of three commercial mouthrinses on extracellular matrix metabolism and human gingival cell behaviour. Toxicol In Vitro. 2016;34:88‐96. 27039991 10.1016/j.tiv.2016.03.015

[25] Carp H , Janoff A . Possible Mechanisms of Emphysema in Smokers. In Vitro Suppression of Serum Elastase‐lnhibitory Capacity by Fresh Cigarette Smoke and its Prevention by Antioxidants 1, 2. Am Rev Respir Dis. 1978;118(3):617‐621. 101105 10.1164/arrd.1978.118.3.617

[26] Pagano S , Lombardo G , Balloni S , et al. Cytotoxicity of universal dental adhesive systems: Assessment in vitro assays on human gingival fibroblasts. Toxicol In Vitro. 2019;60:252‐260. 31195088 10.1016/j.tiv.2019.06.009

[27] ISO . 10993‐5. Biological Evaluation of Medical Devices ‐ Part 5: Tests for In Vitro Cytotoxicity. ISO; 2009. https://www.iso.org/obp/ui/#iso:std:iso:10993:‐5:en. Accessed May 10, 2021.

[28] Pagano S , Lombardo G , Caponi S , et al. Bio‐mechanical characterization of a CAD/CAM PMMA resin for digital removable prostheses. Dent Materials. 2021;37(3):e118‐e130. 10.1016/j.dental.2020.11.003 33257084

[29] Pagano S , Lombardo G , Costanzi E , et al. Morpho‐functional effects of different universal dental adhesives on human gingival fibroblasts: an in vitro study. Odontology. 2021;109(2):524‐539. 33211211 10.1007/s10266-020-00569-x PMC7954759

[30] Chambrone L , Chambrone D , Fe P , Chambrone LA , Lima LA . The influence of tobacco smoking on the outcomes achieved by root‐coverage procedures: a systematic review. J Am Dent Assoc. 2009;140(3):294‐306. 19255173 10.14219/jada.archive.2009.0158

[31] Grafström RC , Norén UG , Zheng X , Elfwing A , Sundqvist K . Growth and transformation of human oral epithelium in vitro. Recent Results Cancer Res. 1997;143:275‐306. 8912427 10.1007/978-3-642-60393-8_20

[32] SchlageWK IAR , Kostadinova R , et al. In vitro systems toxicology approach to investigate the effects of repeated cigarette smoke exposure on human buccal and gingival organotypic epithelial tissue cultures. Toxicol Mech. Methods. 2014;24(7):470‐487. 25046638 10.3109/15376516.2014.943441 PMC4219813

[33] Semlali A , Chakir J , Rouabhia M . Effects of whole cigarette smoke on human gingival fibroblast adhesion, growth, and migration. J Toxicol Environ Health A. 2011;74(13):848‐862. 21598170 10.1080/15287394.2011.570230

[34] Chang SS , Jiang WW , Smith I , et al. Chronic cigarette smoke extract treatment selects for apoptotic dysfunction and mitochondrial mutations in minimally transformed oral keratinocytes. Int J Cancer. 2010;126(1):19‐27. 19634139 10.1002/ijc.24777 PMC2818069

[35] Holliday RS , Campbell J , Preshaw PM . Effect of nicotine on human gingival, periodontal ligament and oral epithelial cells. A systematic review of the literature. J Dent. 2019;86:81‐88. 31136818 10.1016/j.jdent.2019.05.030

[36] Lallier TE , Moylan JT , Maturin E . Greater sensitivity of oral fibroblasts to smoked versus smokeless tobacco. J Periodontol. 2017;88(12):1356‐1365. 28708037 10.1902/jop.2017.170232

[37] Warnakulasuriya KA , Ralhan R . Clinical, pathological, cellular and molecular lesions caused by oral smokeless tobacco–a review. J Oral Pathol Med. 2007;36(2):63‐77. 17238967 10.1111/j.1600-0714.2007.00496.x

[38] Song L , Li J , Yuan X , et al. Carbon monoxide‐releasing molecule suppresses inflammatory and osteoclastogenic cytokines in nicotine‐ and lipopolysaccharide‐stimulated human periodontal ligament cells via the heme oxygenase‐1 pathway. Int J Mol Med. 2017;40(5):1591‐1601. 28901402 10.3892/ijmm.2017.3129

[39] Liu Q , Zhao M , Chen W , et al. Mainstream cigarette smoke induces autophagy and promotes apoptosis in oral mucosal epithelial cells. Arch Oral Biol. 2020;111:104646. 31896026 10.1016/j.archoralbio.2019.104646

[40] Bozkurt SB , Hakki SS . Nicotine suppresses proliferation and mineralized tissue‐associated gene expressions of cementoblasts. J Periodontol. 2020;91(6):800‐808. 31489997 10.1002/JPER.19-0256

[41] Ilies BD , Moosakutty SP , Kharbatia NM , Sarathy SM . Identification of volatile constituents released from IQOS heat‐not‐burn tobacco HeatSticks using a direct sampling method. Tob Control. 2020:tobaccocontrol‐2019‐055521. 10.1136/tobaccocontrol-2019-055521 PMC12171520 32457207

[42] Ahmed HG , Ebnoof SO , Hussein MO , Gbreel AY . Oral epithelial atypical changes in apparently healthy oral mucosa exposed to smoking, alcohol, peppers and hot meals, using the AgNOR and Papanicolaou staining techniques. Diagn Cytopathol. 2010;38(7):489‐495. 19894260 10.1002/dc.21224

[43] Taybos G . Oral changes associated with tobacco use. Am J Med Sci. 2003;326(4):179‐182. 14557730 10.1097/00000441-200310000-00005

[44] Noël A , Verret CM , Hasan F , et al. Generation of Electronic Cigarette Aerosol by a Third‐Generation Machine‐Vaping Device: Application to Toxicological Studies. J Vis Exp. 2018;138:58095. 10.3791/58095 PMC6231858 30199038

[45] Cao X , Muskhelishvili L , Latendresse J , Richter P , Heflich RH . Evaluating the Toxicity of Cigarette Whole Smoke Solutions in an Air‐Liquid‐Interface Human In Vitro Airway Tissue Model. Toxicol Sci. 2017;156(1):14‐24. 28115645 10.1093/toxsci/kfw239

[46] Davis B , To V , Talbot P . Comparison of cytotoxicity of IQOS aerosols to smoke from Marlboro Red and 3R4F reference cigarettes. Toxicol In Vitro. 2019;61:104652. 31526836 10.1016/j.tiv.2019.104652

[47] Korge P , Calmettes G , Weiss JN . Increased reactive oxygen species production during reductive stress: The roles of mitochondrial glutathione and thioredoxin reductases. Biochim Biophys Acta. 2015;1847(6–7):514‐525. 25701705 10.1016/j.bbabio.2015.02.012 PMC4426053

[48] Wang H , Chen L , Zhou T , Zhang Z , Zeng C . Nicotine Promotes WRL68 Cells Proliferation Due to the Mutant p53 Gain‐of‐Function by Activating CDK6‐p53‐RS‐PIN1‐STAT1 Signaling Pathway. Chem Res Toxicol. 2020;33(9):2361‐2373. 32820905 10.1021/acs.chemrestox.0c00119

[49] He L , Chen Y , Feng J , et al. Cellular senescence regulated by SWI/SNF complex subunits through p53/p21 and p16/pRB pathway. Int J Biochem Cell Biol. 2017;90:29‐37. 28716547 10.1016/j.biocel.2017.07.007

[50] Alanazi H , Hj P , Chakir J , Semlali A , Rouabhia M . Comparative study of the effects of cigarette smoke and electronic cigarettes on human gingival fibroblast proliferation, migration and apoptosis. Food Chem Toxicol. 2018;118:390‐398. 29800583 10.1016/j.fct.2018.05.049

[51] Elmore S . Apoptosis: a review of programmed cell death. Toxicol Pathol. 2007;35(4):495‐516. 17562483 10.1080/01926230701320337 PMC2117903

[52] Kim JJ , Khalid O , Duan L , Kim R , Elashoff D , Kim Y . Gene expression signatures affected by ethanol and/or nicotine in normal human normal oral keratinocytes (NHOKs). Genom Data. 2014;2:156‐161. 25126520 10.1016/j.gdata.2014.06.021 PMC4127651

